# Forest soil CO_2_ efflux models improved by incorporating topographic controls on carbon content and sorption capacity of soils

**DOI:** 10.1007/s10533-016-0233-5

**Published:** 2016-08-19

**Authors:** Natalia A. Lecki, Irena F. Creed

**Affiliations:** Department of Biology, Western University, 1151 Richmond Street, London, ON N6A 5B7 Canada

**Keywords:** Forest, Soil, Carbon, Carbon dioxide, Temperature, Moisture, Sorption capacity

## Abstract

Improved models are needed to predict the fate of carbon in forest soils under changing environmental conditions. Within a temperate sugar maple forest, soil CO_2_ efflux averaged 3.58 µmol m^−2^ s^−1^ but ranged from 0.02 to 25.35 µmol m^−2^ s^−1^. Soil CO_2_ efflux models based on temperature and moisture explained approximately the same amount of variance on gentle and steep hillslopes (r^2^ = 0.506, p < 0.05 and r^2^ = 0.470, p < 0.05 respectively). When soil carbon content and sorption capacity were added to the models, the amount of explanation increased slightly on a gentle hillslope (r^2^ = 0.567, p < 0.05) and substantially on a steep hillslope (r^2^ = 0.803, p < 0.05). Within the organic-rich surface of the mineral soil, carbon content was positively related and sorption capacity was negatively related to soil CO_2_ efflux rates. There were general patterns of smaller carbon pools and lower sorption capacity in the upland positions than in the lowland and wetland positions, likely a result of hydrological transport of particulate and dissolved substances downslope, leading to higher soil CO_2_ efflux in the upland positions. However, the magnitude of the soil CO_2_ efflux was mitigated by the higher sorption capacity of the organic-rich surface layer of the mineral soils, which was negatively correlated to soil CO_2_ efflux. More accurate estimates of forest soil CO_2_ efflux must take into account topographic influences on the carbon pool, the environmental factors that affect rates of carbon transformation, as well as the physicochemical factors that determine the fraction of the carbon pool that can be transformed.

**Electronic supplementary material:**

The online version of this article (doi:10.1007/s10533-016-0233-5) contains supplementary material, which is available to authorized users.

## Introduction

Forest soils contain up to 45 % of carbon (C) stored on land surfaces (Malmsheimer et al. 2011) forming an important part of the global C budget (Schlesinger 1997; Hedges et al. 2000). Climate change is expected to have significant consequences on forest soils (Davidson and Janssens 2006; IPCC 2013) and better models are needed to predict current and future soil carbon stocks to help guide in mitigating potential effects that are likely to be associated with climate change. However, quantification of forest soil carbon dioxide (CO_2_) efflux remains a challenge because of spatial heterogeneity at varied scales and temporal variability in the environmental processes that control soil CO_2_ efflux (Emanuel et al. 2011; Chatterjee and Jenerette 2011). Accurate estimates of soil CO_2_ efflux are important in order to determine if the net sink or source status of a forest are changing. We sought to improve these estimates by investigating the effects of topography on the distribution of soil properties that regulate soil CO_2_ efflux.

Several approaches to modelling soil CO_2_ efflux have emerged. The majority of models developed to predict forest soil CO_2_ efflux are fairly simple, accounting for temperature and soil moisture based on their abilities to control the rates of biological reactions in soil microbes (e.g., Kang et al. 2003, 2006; Tang and Baldocchi 2005; Sjögersten et al. 2006; Pacific et al. 2009; Barron-Gafford et al. 2011; Cable et al. 2012; Chang et al. 2014). Temperature is usually the strongest predictor of soil CO_2_ efflux, because temperature directly controls microbial activity and rates of respiration (Davidson et al. 1998, 2006). Indeed, many models of CO_2_ efflux have been created with soil temperature alone (e.g., Chapman and Thurlow 1996; Davidson et al. 1998; Scanlon and Moore 2000). Moisture has a more complex relationship with soil CO_2_ efflux, often inhibiting soil CO_2_ efflux when conditions are too dry or too wet (Welsch and Hornberger 2004; Riveros-Iregui et al. 2007). When conditions are too dry, CO_2_ efflux is limited by the amount of dissolved substrate, and when conditions are too wet, CO_2_ efflux is limited by the amount of dissolved oxygen (Stark and Firestone 1995; Laiho 2006).

Soil CO_2_ efflux from microbial activity (heterotrophic respiration) is also affected by the carbon content (Webster et al. 2008a, b) and carbon sorption capacity [i.e., the ability of dissolved organic carbon (DOC) to sorb to iron (Fe) and aluminum (Al) oxyhydroxides through ligand exchange (Kaiser et al. 1996; Qualls et al. 2002)] of soils. Most research suggests that this sorption renders DOC inaccessible to microbes, thus leading to long-term immobilization of carbon (Kaiser and Guggenberger 2000; Kalbitz et al. 2005; Schneider et al. 2010). However, sorption processes can selectively sorb hydrophobic fractions of dissolved organic matter, with hydrophilic fractions remaining in the dissolved phase (Kaiser and Zech 1998; Ussiri and Johnson 2004). Further, the sorption capacity of fresh mineral surfaces is generally exhausted within several decades and thus the mean residence time for sorbed DOC would follow similar lengths (Guggenberger and Kaiser 2003; Mikutta et al. 2006). This short residence time suggests that at least a portion of the DOC may not undergo long-term immobilization when sorbed to mineral surfaces, and therefore both mobile DOC and sorbed DOC may contribute to soil CO_2_ efflux (Creed et al. 2013).

Topography has long been known to influence carbon pools in soils (Milne 1936). Soils develop in response to hillslope processes, which represent an interplay between static factors (such as elevation, slope and aspect that influence the radiation, temperature and moisture of the soils), and dynamic factors (such as the relative position of the soils along the hillslope, which influences the transport of particulate and dissolved materials downslope) (Young 1972, 1976). Over the past 50 years, this fundamental understanding of how topography influences soils has been transformed by computer digital terrain analysis techniques that can represent both static and dynamic factors that influence soil formation. Recently, Webster et al. (2011) developed a suite of digital terrain analysis techniques to create a template based on topographic positions that reflect distinct geomorphological, hydrological and biogeochemical processes that influence carbon pools and fluxes and that enable upscaling from individual sites (Webster et al. 2008a) to entire watersheds (Webster et al. 2008b). In doing so, they were able to improve substantially watershed-aggregated estimates of soil carbon pools by considering all topographic positions rather than only the dominant topographic position (Webster et al. 2011).

We hypothesize that topography also influences the downward transport of Fe and Al oxyhydroxides, reducing the size of the “microbe accessible” carbon pool in the lowest reaches of hillslopes. In this study, we predict that sorption capacity acts as a sink (negative coefficient) in soil CO_2_ efflux models. We developed soil CO_2_ efflux models for gentle and steep hillslopes using estimates of soil carbon pools, sorption capacity, and the environmental conditions that increase the rate of transformation of mobile (and possibly sorbed) carbon into CO_2_ along the hillslope (soil temperature and moisture). The models were developed from data collected in a sugar maple forest in the Great Lakes-St. Lawrence Forest region of central Ontario, Canada under climatic extremes occurring over the past 30 years. Although heterotrophic respiration may account for as little as 10 % (or as much as 90 %) of annual or growing season total respiration in forest soils, with autotrophic respiration accounting for similar and inverse proportions (Hanson et al. 2000), the model parameters included for comparison in this study relate directly to microbial activities. For this reason, we designed methods to attempt to limit efflux measurements to sources of heterotrophic respiration; subsequently, results and discussions are limited to this component.

## Study area

**Fig. 1:**
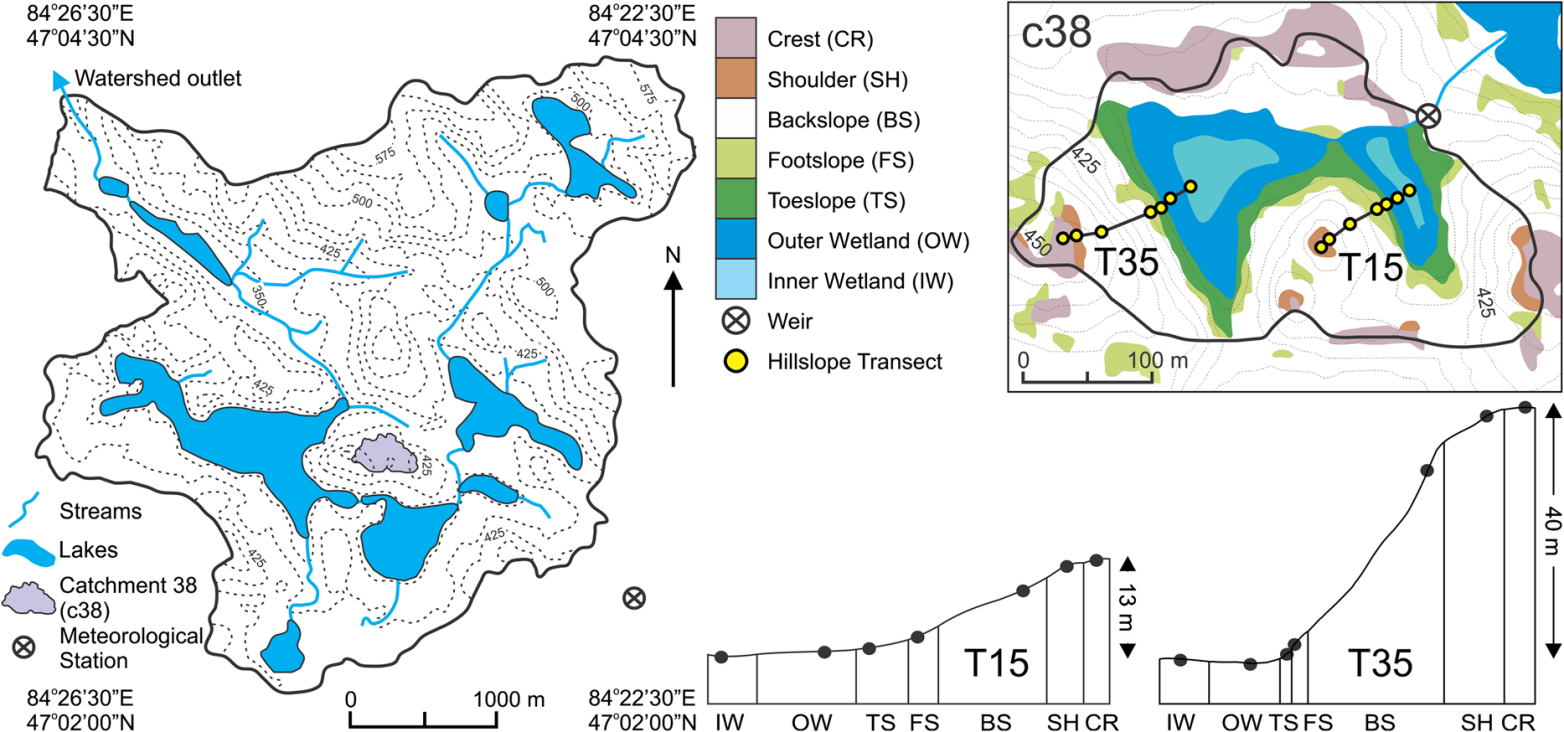
The Turkey lakes watershed and the two experimental hillslopes in catchment 38: the gentle-sloped T15 and the steep-sloped T35

The Turkey lakes watershed (47°03′00″N and 84°25′00″W) is located in the Algoma Highlands of Central Ontario, 60 km north of Sault Ste. Marie and near the eastern shore of Lake Superior (Fig. 1). The climate is continental and strongly influenced by the close proximity to Lake Superior, with a mean annual precipitation of 1189 mm and mean annual temperature of 4.6 °C from 1981 to 2010 (Table 1). The 10.5 km^2^ watershed sits on the northern edge of the Great Lakes-St. Lawrence forest region and consists of an uneven-aged, mature to over-mature, old-growth hardwood system that is >90 % sugar maple. Elevation in the watershed ranges from 644 m above sea level at the summit of Batchawana Mountain to 244 m above sea level at the outlet to the Batchawana River, producing both substantial topographic relief and topographic flats/depressions. The watershed is underlain by Precambrian silicate greenstone, which in turn is overlain by a thin and discontinuous glacial till. The depth of the till ranges from <1 m at higher elevations to 1–2 m at lower elevations. The soils that have developed from these tills are ferro-humic and humo-ferric podzols. Highly organic soils can be found in depressions and adjacent to streams and lakes. The Turkey lakes watershed is a long-term experimental watershed that has been operated by federal government agencies since 1980 (Jeffries et al. 1988).

**Table 1:** Meteorological conditions in the Turkey lakes watershed throughout the year and during the snow free season (April 1 to November 30) over the past 30 years and in 2005 and 2010

	30-year average	2005	2010
Average annual temperature (°C)	4.6	6.0	5.7
Average snow free season temperature (°C)	10.5	12.6	11.5
Total annual precipitation (mm)	1189	930	1024
Total snow free season precipitation (mm)	856	688	894

## Methods

### Experimental design

The study catchment (c38) is 6.33 ha and has a single wetland covering 25 % of the catchment area (1.58 ha). Terrain in the catchment was classified into distinct topographic positions, including inner wetland (IW), outer wetland (OW), toeslope (TS), footslope (FS), backslope (FS), shoulder (SH), and crest (CR) (Fig. 1).

The positions were delineated using digital terrain analysis methods described in Webster et al. (2011) from a 5-m digital elevation model (DEM) interpolated from light detection and ranging (LiDAR) data (horizontal accuracy of 0.15 m under open canopy and 0.30 m under closed canopy). Wetland positions were defined using a probabilistic approach to determine the likelihood of a DEM grid cell being flat or in a depression (Lindsay and Creed 2006). A ground-based survey was used to determine the boundary of the IW, defined as the portion of the wetland with peat depths greater than about 50 cm (deepest depths reached about 5 m), with the remaining area adjacent to the IW but within the delineated wetland classified as the OW. For the hillslope positions, five topographic attributes were derived at each DEM grid cell location including: (1) percent height relative to local pits and peaks from the DEM with pits removed; (2) percent height relative to local channels and divides from the DEM with multiple grid cells that formed depressions removed; (3) slope gradient; (4) slope curvature; and (5) topographic wetness index (Beven and Kirkby 1979) calculated using the infinite direction (Dinf) flow algorithm (Tarboton 1997). For each position, the topographic attributes were converted to fuzzy membership scores between 0 (no probability of being in a given position) and 1 (full probability). The fuzzy scores for each topographic attribute were then combined to assign the probability of a grid cell belonging to each of the positions. The grid cells were assigned the position with the highest probability (c.f. Webster et al. 2011).

Two north-facing hillslope transects were plotted from the wetland to CR positions, one with a relatively gentle slope (T15; 15°) and one relatively steep (T35; 35°). Plots were established within each topographic position along each transect to sample soil chemical properties and monitor soil environment and CO_2_ efflux. Data from 2 years (2005 and 2010) were used to provide a contrast in climate conditions. The 2005 snow free season (April 1 to November 30) was relatively warm (12.6 °C) and dry (688 mm) compared to the 30-year average (10.5 °C and 856 mm, respectively), while the 2010 snow free season was relatively cooler (11.5 °C) and wetter (894 mm) than 2005 (Table 1). Hydrologic periods were defined by precipitation patterns, temperature fluctuations, and water table depths in 2005 and 2010 (Table 2).

**Table 2:** Description of annual snow free season hydrologic periods in the Turkey lakes watershed

Name	Dates	Characteristics
Spring snowmelt	April 1–May 30	Snowmelt, rising temperatures, first drop in water table
Summer	June 1–July 31	Further increase in temperature, variable precipitation, second drop in water table
Late summer	August 1–September 19	Peak temperatures, variable precipitation, possibility of drought, lowest water table depths
Fall storms	September 20–October 25	Decline in temperatures, onset of fall storms, large spikes in precipitation, rapid rise in water table depth
Late fall	October 26–November 30	Further decline in temperatures, little precipitation, water table remains near surface

### Soil CO_2_ efflux

Soils along the hillslopes were instrumented to measure CO_2_ efflux using a ground-based chamber method (Livingston and Hutchinson 1995). Square aluminum collars (0.21 m^2^) were placed within each plot and inserted 10–20 cm into the soil. The collars were allowed to settle for at least one snow free season to minimize disturbance related CO_2_ pulses. Small understory plants were clipped and seeds removed from within the collars 24 h before gas sampling to minimize the effects of aboveground respiration (Webster et al. 2008a). A portable acrylic chamber was inverted over the collars, and the edges were immersed in water to ensure a tight seal. A fan positioned in the top of the chamber ensured equal mixing of the air for the Vaisala CARBOCAP^®^ Carbon Dioxide Probe GMP343 infrared gas analyzer (IRGA). The IRGA was attached to a handheld MI-70 control unit that allowed for compensation of oxygen concentration (20.95 %) and air pressure, as well as a secondary sensor for real-time temperature and humidity correction. Fluxes were calculated as the slope of a linear regression of increasing CO_2_ concentration in the chambers with time. Fluxes were adjusted for the volume of the chamber (dimensions of 49.5 cm × 49.5 cm × 40 cm = 90.2 L), volume of the collar and changes in surface topography within the chamber, and were then volume-corrected based on ambient air temperature and pressure. Soil CO_2_ efflux was measured once between 10 a.m. and 2 p.m. at approximately daily intervals during spring melt (April), semi-weekly intervals during the autumn period of storms, weekly intervals during the early and late growing season, and every 2–3 weeks during the summer.

### Environmental drivers of soil CO_2_ efflux

Soils along the hillslopes were instrumented with temperature and moisture probes at 5 cm below the surface of the mineral horizon at each sample site that were connected to Campbell Scientific CR10X data loggers via an AM16/32 relay multiplexer and powered by batteries that were charged by a 30 W solar panel. Soil temperature was measured with thermocouples constructed using thermocouple wire (Type T Omega FF-T-24-TWSH) and embedded into a 10 cm by 0.635 cm I.D. copper tube with epoxy. Soil moisture was measured with a Campbell Scientific CS616 Water Content Reflectometer (WCR, Campbell Scientific Canada Corp., Edmonton, AB) and converted to volumetric water content based on calibration equations provided by the manufacturer for upland soils and provided by Yoshikawa et al. (2004) for wetland soils. Data logger recorded mean hourly values were averaged for each day. Daily data were not continuous due to logger malfunctions. Regressions were developed to interpolate missing data by correlating existing data with logger data collected from equivalent positions at an adjacent transect throughout the snow free season. All regressions had r^2^ values greater than 0.700 and p values smaller than 0.05.

### Substrate limitation to soil CO_2_ efflux

Substrate samples were collected at all topographic positions from freshly fallen leaves (FFL), the litter-fibric-humic (LFH) layer, and the top 10 cm of soils. FFL samples were collected on 30 cm × 30 cm mesh placed on the surface of forest floor prior to leaf fall and collected prior to the development of a snowpack. LFH layer samples were collected by cutting 15.5 cm × 15.5 cm blocks into the forest floor. Organic soil samples in the wetland were collected using the Jeglum sampler (Jeglum et al. 1992), and the mineral soil samples at TS, FS, BS, SH and CR were collected for chemistry using an open-sided sampler (40 cm × 4.4 cm I.D.) and for bulk density using a split core sampler (32 cm × 4.8 cm I.D.). Mineral soil cores were then subdivided into the organic-rich surface Ah horizon and the eluviated Ae horizon as defined in the Canadian System of Soil Classification (Soil Classification Working Group 1998); these horizons are approximately equivalent to the A and E horizons as defined by the US Department of Agriculture (Soil Survey Staff 1994). Organic soils were treated as Ah. The soil samples were then placed in labeled plastic bags and transported in coolers.

Substrate samples were then analysed for carbon pools and sorbed DOC that was estimated by Fe and Al oxyhydroxide concentrations (Creed et al. 2013). Substrate samples for chemical analysis were dried at 25 °C, and for bulk density at 60 °C for FFL, LFH and organic soil or 105 °C for mineral soil. Soil carbon concentrations were determined using a Carlo-Erba NA2000 analyzer (Milan, Italy). Soil carbon pools in FFL were calculated by multiplying carbon concentrations by leaf mass. Soil organic carbon pools (g m^−2^) in LFH and the A horizon or peat were calculated by multiplying the organic carbon concentration (g g^−1^) by bulk density (g m^−3^) and then by depth (m). Soil carbon pools in the peat were limited to the top 10 cm below the LFH; previous work in this catchment showed that soil CO_2_ efflux from wetland soils drops precipitously from the surface with depth, with most efflux occurring in the top 10 cm, even under drought conditions (Webster et al. 2014). Fe and Al oxyhydroxide concentrations were determined using an ammonium oxalate (AO) extraction and a dithionite-citrate-bicarbonate (DCB) extraction. These extractions allowed for the isolation of poorly crystalline, amorphous, and organically bound Fe and Al, and crystalline Fe (Shaw 2001). Iron and Al oxyhydroxides were analyzed using inductively coupled plasma atomic emission spectroscopy (ICP-AES). Sorption capacity (SC) was determined by the sum of AlAO (Al extracted using AO) and FeD (Fe extracted using DCB). For further details, refer to Creed et al. (2013).

### Statistical analysis and modeling

The influence of topographic positions within a given transect on soil CO_2_ efflux, environmental (temperature and moisture) and substrate (carbon pools and sorption capacity) variables was analyzed using ANOVAs on Ranks with post hoc Dunn’s tests, and the influence of the gentle sloped T15 versus steep sloped T35 was analyzed using t-tests or Mann–Whitney U tests (as appropriate).

Soil CO_2_ efflux (μmol m^−2^ s^−1^) was modeled as an exponential relationship, with the exponent a polynomial expression that is linear with respect to temperature and quadratic with respect to moisture (Tang and Baldocchi 2005):

$${\text{CO}}_{2}\;{\text{efflux}} = \exp \left( {a_{1} + a_{2} {\text{T}} + a_{3} {\text{M}} + a_{4} {\text{M}}^{2} } \right)$$

where *a*_i_ are coefficients, T is temperature (°C) and M is moisture (volume%). Linear offsets were added to this exponential relationship to evaluate the effects of adding substrate properties (i.e., carbon pools and sorption capacity) to soil CO_2_ efflux model performance:

$$\begin{aligned} {\text{CO}}_{2}\;{\text{efflux}} &= \exp \left(a_{1} + a_{2} {\text{T}} + a_{3} {\text{M}} + a_{4} {\text{M}}^{2} + a_{5} {\text{C}}_{\text{FFL}} \right.\\&\left.\quad\quad\quad+\, a_{6} {\text{C}}_{\text{LFH}} + a_{7} {\text{C}}_{\text{Ah}} + a_{8} {\text{C}}_{\text{Ae}} \right)\end{aligned}$$

$$\begin{aligned} {\text{CO}}_{2}\;{\text{efflux}} &= \exp ( a_{1} + a_{2} {\text{T}} + a_{3} {\text{M}} + a_{4} {\text{M}}^{2} \\& \quad+ a_{5} {\text{SC}}_{\text{Ah}} + a_{6} {\text{SC}}_{\text{Ae}} ) \end{aligned}$$

$$\begin{aligned} {\text{CO}}_{2}\;{\text{efflux}} &= \exp ( a_{1} + a_{2} {\text{T}} + a_{3} {\text{M}} + a_{4} {\text{M}}^{2} \\&\quad\quad\quad a_{5} {\text{C}}_{\text{FFL}} + a_{6} {\text{C}}_{\text{LFH}} + a_{7} {\text{C}}_{\text{Ah}} + a_{8} {\text{C}}_{\text{Ae}}\\ &\quad\quad\quad+ a_{9} {\text{SC}}_{\text{Ah}} + a_{10} {\text{SC}}_{\text{Ae}} ) \end{aligned}$$

where C is carbon pool content (g C m^−2^) and SC is carbon sorption capacity (mol m^−2^) The best model was considered the model with the lowest Akaike Information Criterion value (AICc) (Webster et al. 2009). The AICc measures the relative quality of models with a bias correction that accounts for the inflation of explained variance in models with larger numbers of parameters (Burnham and Anderson 2002); this criterion is appropriate when evaluating models with different numbers of parameters. Linear regression was performed on the studentized residuals of the best model to determine if there were consistent over- or under-estimates of CO_2_ efflux. Statistical analysis and modeling were performed using SigmaPlot 12.0 (SysStat Software Inc. 2008), and a p value of 0.05 was used to determine significance of all statistical tests.

## Results

### Soil CO_2_ efflux

**Fig. 2:**
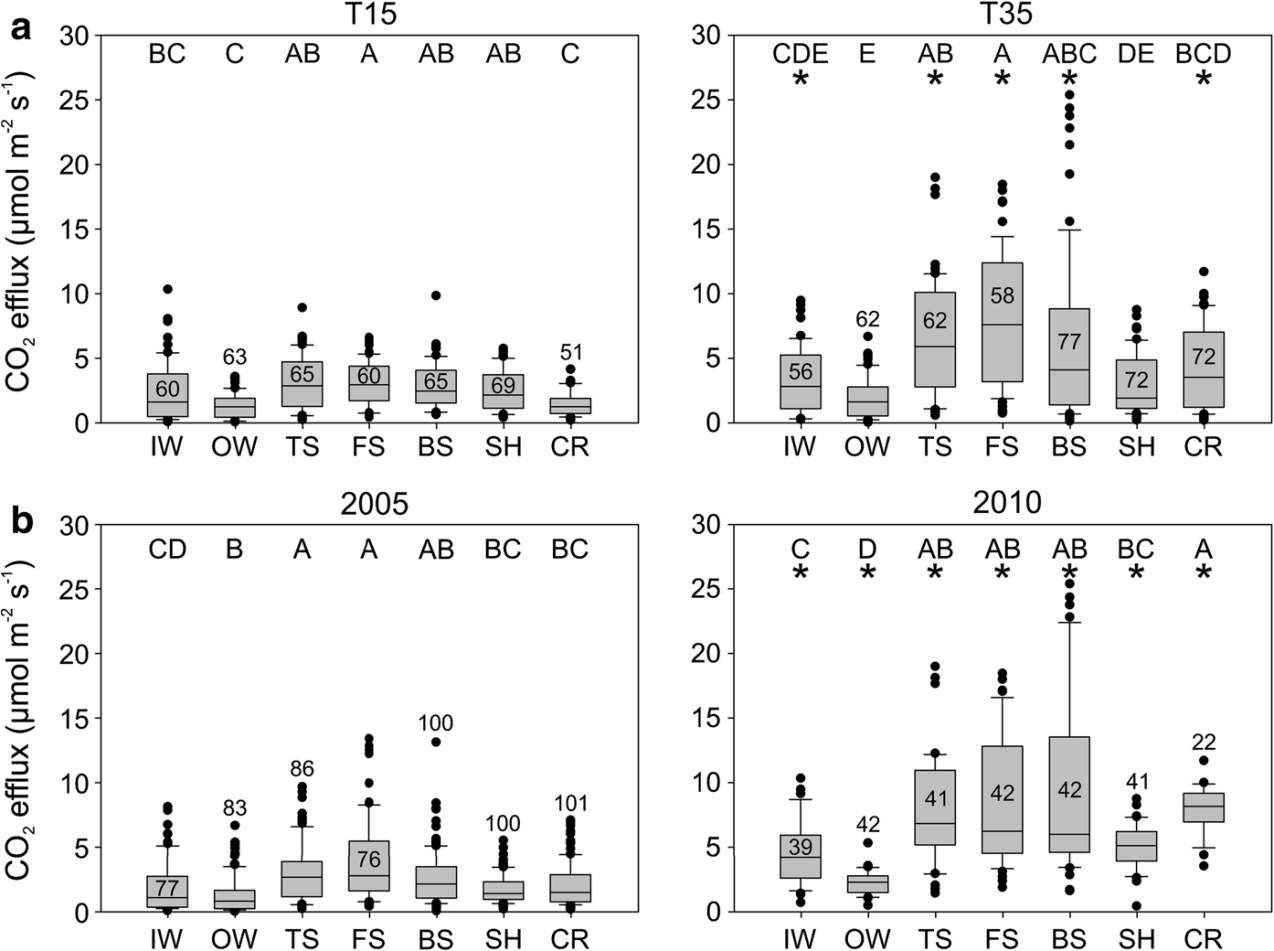
Soil CO_2_ efflux box plots across topographic positions for **a** different hillslopes [the gentle T15 (*left*) and steep T35 (*right*)] and **b** different years [the relatively warm and dry 2005 (*left*) and the relatively cool and wet 2010 (*right*)]. *Different letters* indicate statistically significant differences (p < 0.05) among topographic positions within a hillslope. An *asterisk* indicates this topographic position has significantly more soil CO_2_ efflux than the same position in the other hillslope. *Numbers* indicate sample sizes for each topographic position

Soil CO_2_ efflux over the sampling periods averaged 3.58 µmol m^−2^ s^−1^ and ranged from 0.02 to 25.35 µmol m^−2^ s^−1^. There were significant differences in efflux among topographic positions on both T15 (p < 0.05) and T35 (p < 0.05) (Fig. 2a). Median soil CO_2_ efflux was highest at TS, FS and BS (3.54, 4.27 and 3.27 µmol m^−2^ s^−1^ respectively), and among the lowest at IW and OW (1.97 and 1.40 µmol m^−2^ s^−1^ respectively). Soil CO_2_ efflux was significantly lower at all positions on T15 compare to T35, except at OW and SH, where there was no significant difference (p < 0.05 for all comparisons with significant differences; Fig. 2a). There were also significant differences during and between 2005 (p < 0.05) and 2010 (p < 0.05) (Fig. 2b). Soil CO_2_ efflux was significantly lower at all positions in the relatively warm, dry 2005 compared to the cooler and wetter 2010 (p < 0.05 for all comparisons; Fig. 2b).

### Soil environment

**Fig. 3:**
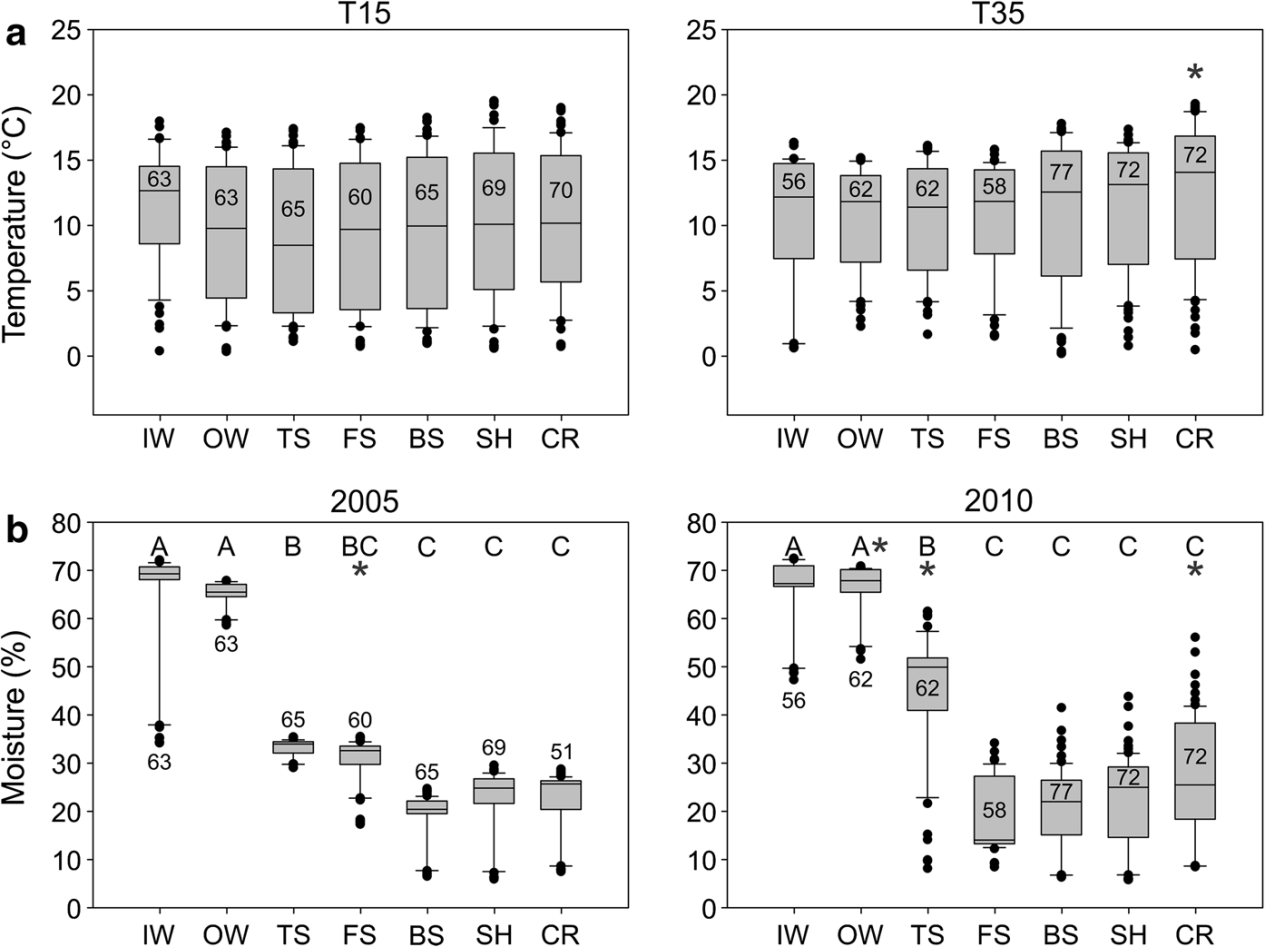
*Boxplots* of **a** soil temperature and **b** soil moisture by topographic position on the relatively gentle T15 (*left*) and steep T35 (*right*) hillslopes. There was no significant difference in soil temperature across topographic positions, but there were significant differences in soil moisture across positions. *Different letters* indicate significant differences (p < 0.05) among topographic positions within a hillslope. *Asterisk* indicates this topographic position has significantly larger temperature or moisture than the same position on the other hillslope. Sample sizes are indicated for each topographic position

Soil temperature averaged 10.34 °C and ranged from 0.16 to 19.51 °C, while soil moisture averaged 36.82 % and ranged from 5.72 to 72.42 %. There were no significant differences in soil temperature among topographic positions on T15 (p = 0.312). There were significant differences in soil temperature among topographic positions on T35 (p < 0.05) but no significant differences in any pairwise comparisons between positions. Soil temperature at CR was significantly different between the two hillslopes (median of 10.18 °C on T15 compared to 14.07 °C on T35, p < 0.05) (Fig. 3a).

Soil moisture was more heterogeneous among the topographic positions of both hillslopes (p < 0.05; Fig. 3b). Highest soil moisture was observed at wetland positions (medians of 69.28 % at IW and 65.50 % at OW on T15; and medians of 67.25 % at IW and 67.86 % at OW on T35). Lowest soil moisture was observed at BS, SH and CR (medians of 20.43, 24.83 and 25.69 % respectively on T15; 22.00, 25.00 and 25.48 % respectively on T35). Intermediate soil moisture was observed at TS and FS (medians of 33.97 and 32.59 % respectively on T15; 49.91 and 14.04 % respectively on T35) (Fig. 3b). OW, TS and CR were drier on T15 than on T35 (p < 0.05), FS was wetter (p < 0.05), and there were no significant differences at BS and SH (p = 0.052 and p = 0.22 respectively).

### Soil carbon pools

**Fig. 4:**
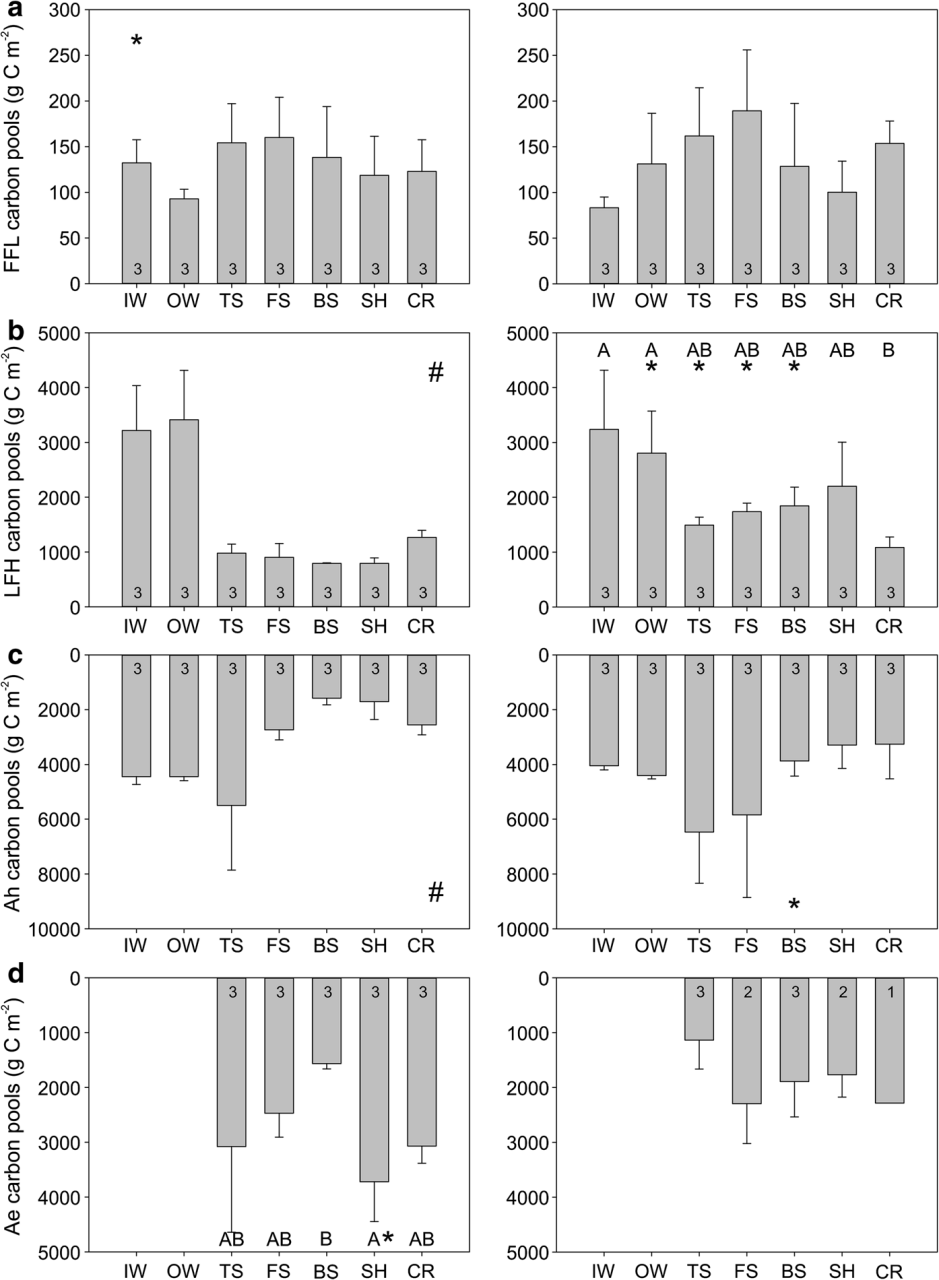
Average carbon pools (g C m^−2^) by topographic position in the **a** freshly fallen leaves (FFL), **b** litter-fibric-humic (LFH), **c** Ah and **d** Ae soil layers for the gentle T15 (*left*) and steep T35 (*right*) hillslopes. *Different letters* indicate significant differences (p < 0.05) among topographic positions within a hillslope. An *asterisk* indicates this topographic position has significantly larger carbon pools than the same position on the other hillslope. A *pound sign* indicates the ANOVA on ranks was significant, but post hoc tests were not able to detect a significant difference among the topographic positions. Sample sizes are indicated for each topographic position

Soil carbon pools in FFL, LFH, Ah and Ae were heterogeneous but showed no systematic pattern within or between hillslopes (Fig. 4).

Soil carbon pools in the FFL layer averaged 131.66 g C m^−2^, ranged from 88.39 to 185.85 g C m^−2^, but had no significant differences among topographic positions within each hillslope (p = 0.26 on T15, p = 0.21 on T35) (Fig. 4a). There was a significant difference in the FFL layer at IW between the hillslopes, with IW having more carbon on T15 than T35 (medians of 135.56 vs. 89.38 g C m^−2^, p < 0.05).

Soil carbon pools in the LFH layer averaged 1824.75 g C m^−2^, ranged from 791.71 to 3413.13 g C m^−2^, and had significant differences among topographic positions on both hillslopes based on ANOVA (p < 0.05; Fig. 4b) but no significant differences between individual topographic positions on T15 based on post hoc tests. Soil carbon pools were largest in IW and OW (medians of 3216.80 and 3413.14 g C m^−2^ respectively on T15; 3237.61 and 2805.07 g C m^−2^ respectively on T35) and significantly larger in IW and OW compared to CR on T35 (median of 1084.43 g C m^−2^ at CR; p < 0.05). There was more LFH carbon in TS, FS, BS and SH on T35 (medians of 1492.36, 1737.39, 1843.56 and 2200.36 g C m^−2^ respectively) compared to T15 (medians of 902.54, 792.18, 791.71 and 1267.10 g C m^−2^ respectively; p < 0.05).

Soil carbon pools in the Ah horizon averaged 3875.37 g C m^−2^, ranged from 1578.32 to 6471.74 g C m^−2^, and had significant differences among topographic positions on T15 (p < 0.05) but no significant differences between individual topographic positions on either hillslope based on post hoc tests (Fig. 4c). No significant differences were found among topographic positions on T35 (p = 0.10). The only significant difference between hillslopes was a larger carbon pool in BS on T35 than T15 (3868.59 vs. 1578.3 g C m^−2^, p < 0.05).

Soil carbon pools in the Ae horizon averaged 2864.20 g C m^−2^ and ranged from 1136.43 to 4442.20 g C m^−2^. There were no significant differences among topographic positions on T35 (p = 0.24; Fig. 4d). There were significant differences on T15 (p < 0.05), with significantly more Ae carbon at the SH compared to at the BS (medians of 3720.28 vs. 1565.47 g C m^−2^ respectively; p < 0.05). There was generally more Ae carbon on T15 compared to T35, though only significantly more in the SH position (3720.28 g C m^−2^ on T15 vs. 1768.13 g C m^−2^ on T35, p < 0.05).

### Soil sorption capacity

**Fig. 5:**
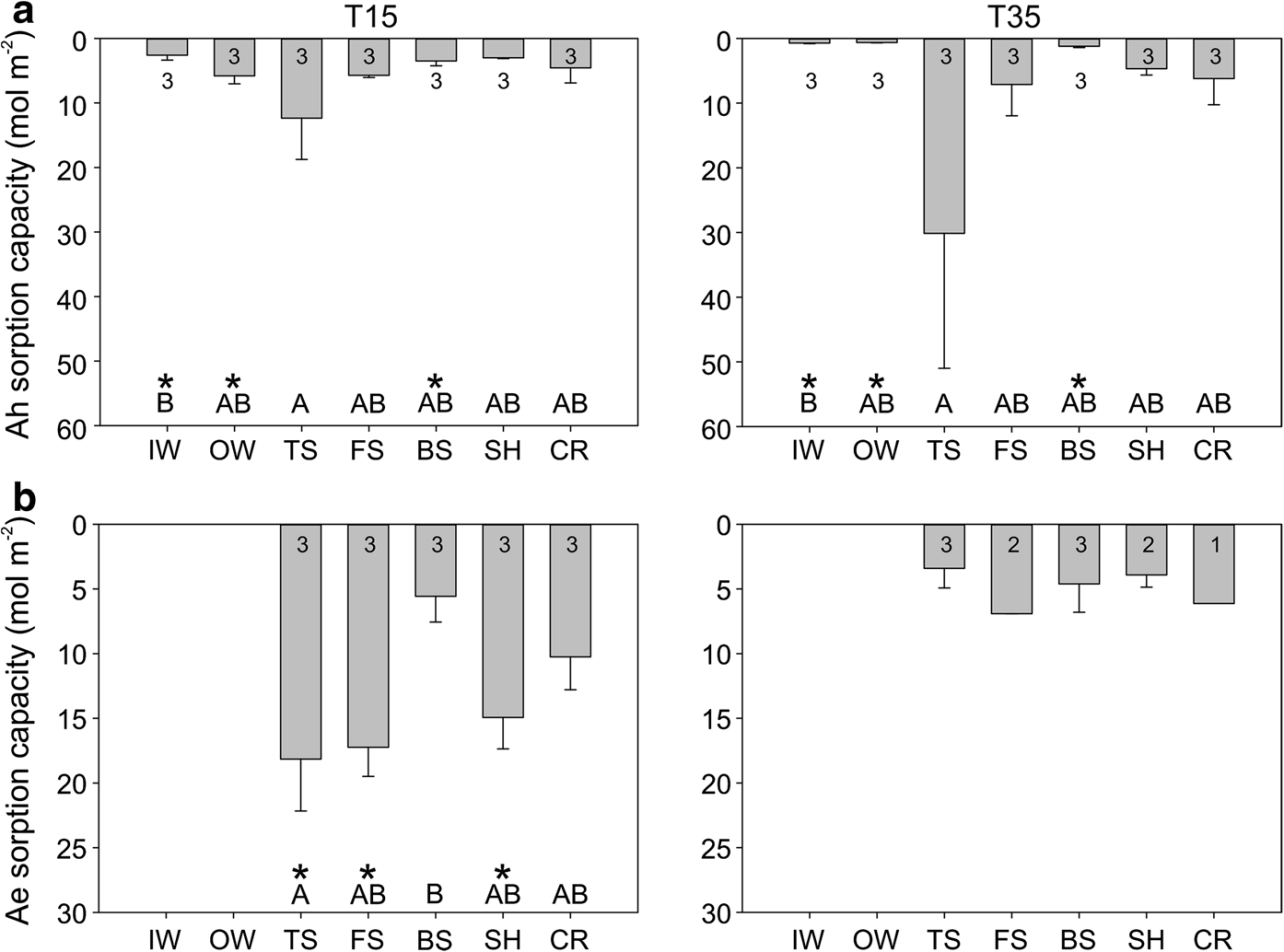
Average sorption capacity (mol m^−2^) by topographic position in the **a** Ah and **b** Ae soil layers for the gentle T15 (*left*) and steep T35 (*right*) hillslopes. *Different letters* indicate significant differences (p < 0.05) among topographic positions within a hillslope. An *asterisk* indicates this topographic position has significantly larger sorption capacity than the same position on the other hillslope. Sample sizes are indicated for each topographic position

Soil sorption capacity was largest in the depositional positions below the steepest portions of the hillslopes, and the sorption capacity was larger in the depositional position below the steeper slope than the gentle slope, but the rest of the gentle slope typically had larger sorption capacities than the steeper slope (Fig. 5).

Soil sorption capacity in the Ah horizon averaged 6.20 mol m^−2^ and ranged from 0.63 to 30.13 mol m^−2^. There were significant differences among topographic positions on both T15 and T35 (p < 0.05) (Fig. 5a), but no significant differences between hillslopes. The TS position had the largest sorption capacity on both hillslopes (medians of 12.35 mol m^−2^ on T15 and 30.13 mol m^−2^ on T35). Sorption capacity was larger at IW, OW and BS on T15 (medians of 2.59, 5.80 and 3.50 mol m^−2^ respectively on T15; and 0.72, 0.63 and 1.21 mol m^−2^ respectively on T35; p < 0.05), but smaller at SH (3.01 mol m^−2^ on T15 vs. 4.19 mol m^−2^ on T35; p < 0.05).

Soil sorption capacity in the Ae horizon averaged 7.18 mol m^−2^ and ranged from 0.63 to 18.15 mol m^−2^. There were significant differences among topographic positions on T15 (p < 0.05) with sorption capacity in the Ae horizon significantly larger at TS and FS than at BS (medians of 18.15, 17.24 and 5.57 mol m^−2^ respectively, p < 0.05) and larger at TS than at CR (median of 10.25 mol m^−2^, p < 0.05) but there were no significant differences on T35 (p = 0.20) (Fig. 5b). Sorption capacity in the Ae horizon was larger on T15 at TS, FS and SH than on T35 (medians of 18.15, 17.24 and 14.93 mol m^−2^ respectively on T15; and 3.41, 6.91 and 3.91 mol m^−2^ respectively on T35; p < 0.05).

### Soil CO_2_ efflux models

The soil CO_2_ efflux model based on temperature and moisture at all topographic positions on both hillslopes explained 38.3 % of the variance in soil CO_2_ efflux based on all 892 samples (Table 3). The inclusion of carbon pools (proxy for substrate quantity) improved model performance by explaining 63.0 % of the variance, whereas the inclusion of sorption capacity (proxy for sorbed DOC) improved model performance by explaining 49.6 % of the variance. The combination of carbon pools and sorption capacity resulted in the best model performance based on AICc values (72.2 % of variance explained) (Table 4).

**Table 3:** Soil CO_2_ efflux models by parameter [bold numbers indicate that the parameters were significant contributors to the models (p < 0.05)]

Model	Parameters	r^2^	Adj. r^2^	AICc Value	Significant C or SC Pools
All data (n = 892)	T + M	0.383	0.381	1875.1	
	T + M + C	0.630	0.627	1501.5	Ah, Ae
	T + M + SC	0.496	0.493	1715.0	Ah, Ae
	T + M + C + SC	**0.722**	**0.720**	**1270.5**	**C: LFH, Ah** **SC: Ah, Ae**
T15 (n = 433)	T + M	0.506	0.502	209.6	
	T + M + C	0.543	0.535	184.2	Ae
	T + M + SC	0.514	0.509	206.3	Ah, Ae
	T + M + C + SC	**0.567**	**0.558**	**164.8**	**C: FFL, LFH, Ah, Ae** **SC: Ah**
T35 (n = 459)	T + M	0.470	0.466	1086.8	
	T + M + C	0.704	0.700	826.6	LFH, Ah
	T + M + SC	0.518	0.513	1047.1	Ah, Ae
	T + M + C + SC	**0.803**	**0.799**	**644.6**	**C: FFL, LFH, Ah** **SC: Ah**

**Table 4:** Regression coefficients and diagnostics of soil CO_2_ efflux models [bold numbers indicate that parameters were significant contributors to the models (p < 0.05)]

		All data			T15			T35		
		Co-efficient	Std. error	p	Co-efficient	Std. error	p	Co-efficient	Std. error	p
Intercept		**−1.5983**	0.288	<0.05	**1.2463**	0.700	<0.05	**−2.4532**	0.425	<0.05
T		**0.1603**	0.006	<0.05	**0.0945**	0.006	<0.05	**0.1918**	0.007	<0.05
M		**0.0519**	0.005	<0.05	**0.0437**	0.010	<0.05	**0.0563**	0.005	<0.05
M^2^		**−0.0008**	<0.001	<0.05	**−0.0006**	<0.001	<0.05	**−0.0007**	<0.001	<0.05
FFL	C	−0.0019	0.001	0.10	**−0.0072**	0.004	<0.05	**−0.0317**	0.003	<0.05
LFH	C	**−0.0006**	<0.001	<0.05	**−0.0009**	<0.001	<0.05	**−0.0008**	<0.001	<0.05
Ah	C	**0.0006**	<0.001	<0.05	**0.0006**	<0.001	<0.05	**0.0015**	<0.001	<0.05
Ae	C	0.0001	<0.001	0.19	**−0.0004**	<0.001	<0.05	−0.0002	<0.001	0.51
Ah	SC	**−0.05**	0.005	<0.05	**−0.2031**	0.047	<0.05	**−0.0938**	0.008	<0.05
Ae	SC	**−0.1001**	0.009	<0.05	0.0232	0.017	0.17	0.2051	0.115	0.074
r^2^		0.690			0.567			0.803		
p		<0.05			<0.05			<0.05		

When soil CO_2_ efflux models were developed for each hillslope, model performance improved substantially. Inclusion of temperature and moisture at all topographic positions resulted in models that explained 50.6 % of the variance on the relatively gentle-sloped T15 (n = 433) and 47.0 % on the variance on the relatively steep-sloped T35 (n = 459) vs. 38.3 % for the combined hillslope model (Table 3). Including substrates to the T15 models did not have a large effect, although the best model was one with both carbon pools and sorption capacity (56.7 % variance explained) (Table 4). In contrast, including substrates to the model for T35 had a much larger effect, and the best model, which included carbon pools and sorption capacity, explained 80.3 % of the variance (Table 4).

In the best models for each hillslope, soil temperature and moisture had significant positive coefficients meaning that higher values of these variables promote higher rates of soil CO_2_ efflux. However, the parameter of moisture squared had significant negative coefficients. Squaring emphasizes extremes in the range of moisture values, suggesting that very large soil moisture values caused by storm events alter the typical moisture control on soil CO_2_ efflux. Carbon pools in the FFL and LFH layers were sinks (negative coefficients) although carbon pools in the FFL layer were a significant sink only in the individual hillslope models (and not the combined hillslope model). In contrast, carbon pools in the Ah horizon were a source (positive coefficient), promoting higher rates of soil CO_2_ efflux. Carbon pools in the Ae horizon were a sink (negative coefficient) on T15, but were not significant on T35 or in the combined hillslope model. Sorption capacity in the organic-rich Ah horizon was a sink (negative coefficient). Sorption capacity in the Ae was also a sink in the combined hillslope model but was not significant in the individual hillslope models. Based on the coefficients, the carbon pool in the Ah horizon was a stronger positive control on T35 than on T15, and sorption capacity in the Ah horizon was a weaker negative control of soil CO_2_ efflux on T35 than on T15. However, the signs of the coefficients of carbon pools and sorption capacity in the Ae horizon were not stable (i.e., were different for the combined hillslope model vs. the individual hillslope models) and it would be difficult to conclude anything about their roles as sinks or sources.

**Fig. 6:**
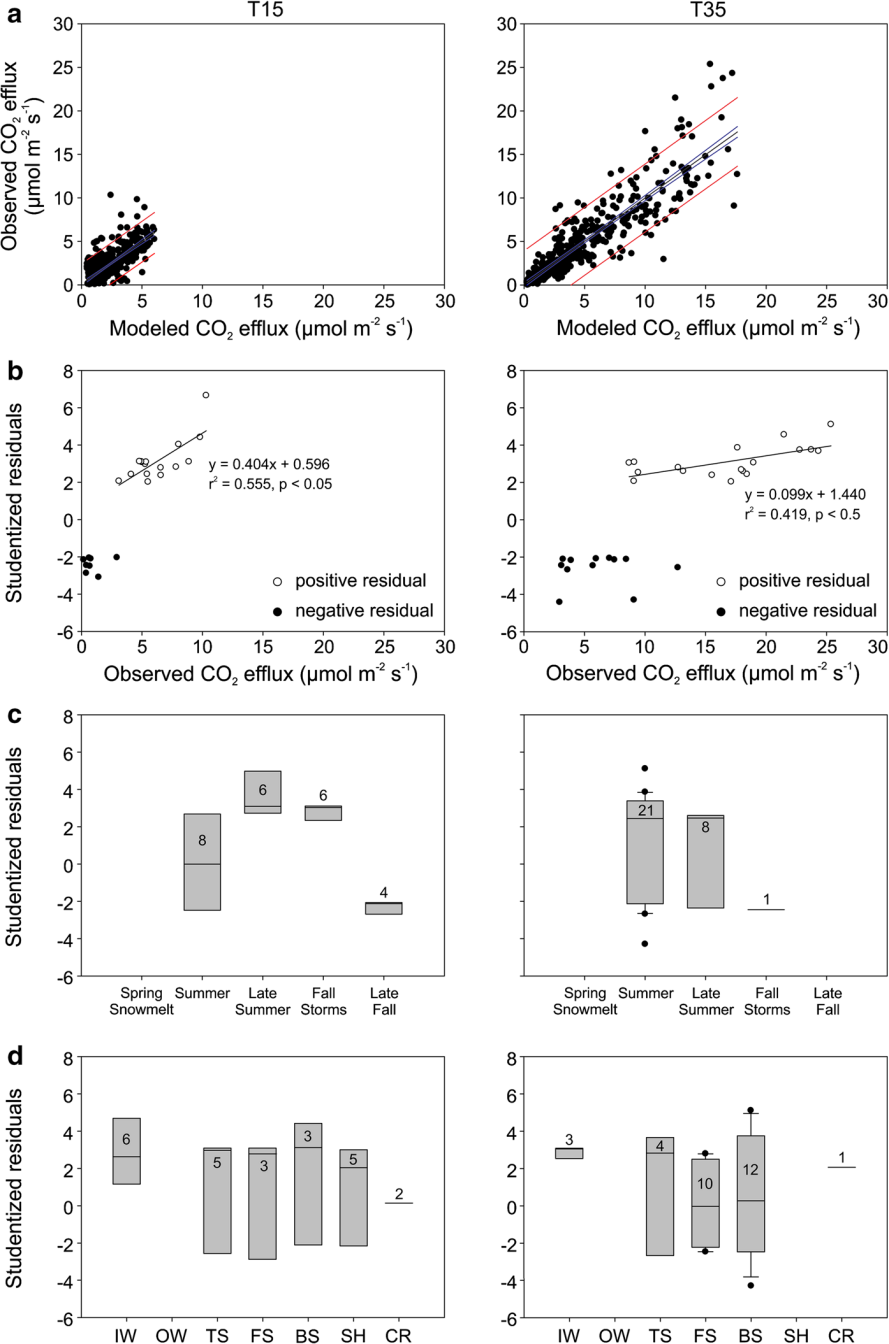
The strength of the best CO_2_ efflux models for the gentle T15 (*left*) and the steep T35 (*right*) hillslopes: **a** Observed soil CO_2_ efflux and modelled soil CO_2_ efflux as a function of soil temperature, soil moisture, carbon pools, and sorption capacity; residuals falling outside the 95 % prediction interval of modelled soil respiration as a function of **b** observed soil CO_2_ efflux (µmol m^−2^ s^−1^), **c** hydrologic period, and **d** topographic position. The number of outliers are indicated for each topographic position and hydrologic period. *Red lines *indicate 95 % prediction intervals

Even the best models had residuals that fell outside the 95 % prediction interval. Observed measurements that fell outside the 95 % prediction interval for modelled soil CO_2_ effluxes were identified for each of the T15 and T35 models (Fig. 6a). For both models, there was a linear relationship between the positive (observed minus predicted) studentized residuals falling outside of the 95 % prediction interval and observed soil CO_2_ efflux (Fig. 6b), showing that the model was more likely to underestimate large soil CO_2_ efflux events. The largest positive residuals occurred during the summer, late summer and fall storm hydrologic periods (Fig. 6c). Large residuals occurred at all topographic positions except the OW, but predominantly at IW, TS, FS, and BS (Fig. 6d).

## Discussion

Soil CO_2_ efflux is most commonly modelled as a function of temperature and moisture (Kang et al. 2003, 2006; Tang and Baldocchi 2005; Sjögersten et al. 2006; Pacific et al. 2009). In this study, we found agreement that temperature and moisture were important drivers of CO_2_ efflux. Topography influences the distribution of soil moisture and temperature on the landscape and, therefore, variability in soil CO_2_ efflux. However, a temperature and moisture regression model that included data from both the gentle (T15) and steep (T35) slopes explained only 38.3 % of variance in soil CO_2_ efflux, which was lower than other studies (e.g., Davidson et al. 1998; Webster et al. 2008a). In particular, Webster et al. (2008a), who conducted similar research in the same study area, explained 57 % of variance in hillslopes spanning the same range in slopes but using data from only 1 year (a relatively warm, dry year). Our study used an additional year of data (2010, a relatively cool, wet year), which substantially reduced the amount of variance explained. However, when we analyzed the hillslopes separately, the percent variance in soil CO_2_ efflux explained using temperature and moisture increased to 50.6 and 47.0 % for the gentle and steep hillslopes respectively. This suggests that while both temperature and moisture are important drivers of soil CO_2_ efflux, their relative contributions differed and responded to the degree of slope at the sampling site. Indeed, the coefficient for temperature was twice as large on T35 as on T15, which suggests that formation of slope-dependent microclimates produces heterogeneous distributions of soil water content among slopes (Kang et al. 2003).

Topography also influences the distribution and quality of substrates on the landscape, with important implications for microbial activities that drive heterotrophic respiration. We found that topography-driven heterogeneity in soil carbon pools and sorption capacity in addition to moisture and temperature had a strong effect on our ability to predict soil CO_2_ efflux. Adding substrates to a model incorporating moisture and temperature improved the explanation of variance by only 6.1 % on T15 from 50.6 to 56.7 %, suggesting that topography has relatively minimal influence on substrate distribution on this gentle slope. In contrast, adding substrates to a model on T35 explained an additional 33.3 % of the variance in CO_2_ efflux from 47.0 to 80.3 %. This suggests that topography has a strong influence on substrate distribution on this steep slope, delivering substrates to environmentally optimal soil CO_2_ production zones (i.e., the BS, FS, and TS positions). Including carbon pools and sorption capacity in soil CO_2_ efflux models is therefore important in areas of more than minimal relief.

In particular, the addition of soil carbon pools increased the explanatory power of soil CO_2_ efflux models, especially on T35 where the amount of variance explained increased from 47.0 to 70.4 % from a model incorporating only moisture and temperature (this compared to an increase from 50.6 to 54.3 % on T15). Water residence time is longer on gentle slopes and shorter on steep slopes, influencing the transport of particulate and dissolved materials downslope. On both hillslopes, the forest floor (FFL and LFH layers) served as a sink for soil CO_2_ efflux, likely because carbon was leached vertically down or laterally to the stream during storms or snowmelt (Davidson and Janssens 2006) and was therefore not available for soil CO_2_ efflux. The FFL layer was more negatively correlated to CO_2_ efflux on the steeper slope; this is possibly the result of a greater redistribution of substrate (DOC) on steeper slopes to lowland (FS, TS) and wetland (OW, IW) positions through less-reactive surface hydrological pathways. Carbon pools in the organic-rich Ah horizon were a source of soil CO_2_ efflux, especially on the steeper T35. Carbon that was mobilized to mineral soils may have become metabolized during shallow subsurface preferential flows that may be more common on steeper slopes (Jobbagy and Jackson 2000). This suggests that the Ah horizon is a reactive pathway more associated with microbial respiration.

The addition of sorption capacity also increased the explanatory power of soil CO_2_ efflux models developed with temperature and moisture, although the amount of additional variance explained in these models was less than with the addition of carbon pools. There were relatively small increases in the amount of variance explained from 50.6 to 51.4 % on T15 and from 47.0 to 51.8 % on T35, but a more substantial increase from 38.3 to 49.6 % when data from both hillslopes were combined. There was a negative relationship between sorption capacity in the organic-rich Ah horizon and soil CO_2_ efflux, confirming for this horizon that sorption capacity acts a sink. DOC is thought to be the primary substrate for microbial soil CO_2_ efflux because it is labile and readily absorbed by microbes (Bengtson and Bengtsson 2007). Several studies have suggested that most DOC within the soil profile is derived from older carbon solubilized from the Ah horizon rather than from the litter layers (Hagedorn et al. 2004; Müller et al. 2009; Kramer et al. 2010). This supports our finding that the organic-rich Ah horizon was where microbes accessed the majority of substrate that, once metabolized, contributed to soil CO_2_ efflux. However, there may be processes that constrain microbial access to this carbon pool. We found that the organic-rich Ah horizon was negatively correlated with soil CO_2_ efflux, suggesting that Fe and Al oxyhydroxides in the Ah horizon were strongly binding DOC (or that microbes in the organic-rich Ah horizon could have been processing DOC rapidly and the sampling was failing to capture the hot moment of CO_2_ efflux). The negative effect of sorption capacity in the organic-rich Ah horizon on soil CO_2_ efflux was smaller on the steeper T35. Steeper slopes may cause higher rates of downslope transport of finer particles with higher DOC binding capacity, as well as higher rates of DOC transport downslope that ensure sufficient substrate (DOC) which more rapidly saturate the binding sites. These processes could therefore have masked the effect of sorption capacity on soil CO_2_ efflux due to the saturation of binding sites that created a DOC supply that could be respired by microbes (Kaiser et al. 1996). Therefore, the topographic controls on the distribution of both carbon pools and sorption capacity must be considered if we are to improve soil CO_2_ efflux model performance.

The analysis of residuals of the soil CO_2_ efflux models (Fig. 6b) indicated systematic underestimates of observed soil CO_2_ efflux as the magnitude of soil CO_2_ efflux increased. The linear relationship in the residuals was strongest (higher r^2^, larger coefficient in the regression equation) on T15, indicating that the model for the gently sloped hillslope underestimated soil CO_2_ efflux to a greater degree than the model for the steeper hillslope. Residuals were mostly positive (i.e., regression models underestimated soil CO_2_ efflux) and occurred most frequently (1) during the late summer/early autumn periods (Fig. 6c), (2) at IW on both hillslopes (positive residuals only), (3) at TS, FS, BS and SH on T15 (both negative and positive residuals but with positive residual medians), and (4) at TS, FS and BS on T35 (both negative and positive residuals with positive residual medians at TS only and near zero at FS and BS) (Fig. 6d).

We designed methods in this study to minimize root respiration of small plants by clipping aboveground vegetation 24 h prior to sampling. Despite this, some respiration from deeper roots may have contributed to efflux measurements; this autotrophic respiration may co-vary with heterotrophic respiration in the presence of soil or substrate qualities that promote both. However, the presence of systematic residuals would also suggest that other factors should be considered to capture the full heterogeneity of soil CO_2_ efflux on forested landscapes. The largest residuals occurred during the late summer/early autumn period, which suggests that surface and near surface conditions were especially important to soil CO_2_ efflux during the drier summer months during rainstorm events. Recent studies have found that rainstorms, especially during peak seasons for soil CO_2_ efflux, result in highly variable pulses of soil CO_2_ efflux (Wu and Lee 2011). For example, the IW position may have been influenced by a hydrological “decoupling” between surface and subsurface processes during a rain event, especially in late summer/early fall when water table depths typically drop well below the surface. It is possible that the rain triggers soil CO_2_ efflux events by creating optimal conditions that include delivery of fresh substrate from rain passing through the canopy to sedentary soil microbes, in addition to providing optimal temperature and moisture conditions for microbial respiration (e.g., Enanga et al. 2016). Further, labile carbon-laden water in the uplands would tend to flow rapidly downslope through surface and shallow subsurface flowpaths during a rain event, increasing carbon pools in the FS and TS positions especially on steeper transects (Riveros-Iregui and McGlynn 2009). The next generation of soil CO_2_ efflux models will need to capture rain-triggered conditions that may lead to large soil CO_2_ efflux events, but will rely on the development of new techniques to measure the magnitude and movement of precursors of soil CO_2_ efflux in the forest floor and the shallow subsurface of forest soils.

## Conclusion

Forest soil CO_2_ efflux models based on topographic controls on soil temperature and moisture are limited in their ability to provide realistic estimates. Adding topographic controls on soil carbon pools significantly improved model performance, but adding the potential for soils to sorb carbon produced the best model performance. Topography results in the downward transport of particulate and dissolved materials of carbon that create areas of high soil CO_2_ efflux at the interface between uplands and wetlands, but it also results in the downward transport of Fe and Al oxyhydroxides to the lowest reaches of the hillslopes that can immobilize carbon, rendering it unavailable for microbial transformation. The greatest improvement in model performance was found by including soil carbon pools and sorption capacity with soil temperature and moisture parameters in a CO_2_ efflux model on a steep hillslope. The steeper the topography, the greater the potential for downward transport of both carbon and carbon sorbing substances, especially the organic-rich surface of the mineral soil, pointing to potential vulnerable areas of the forest landscape that may produce major soil CO_2_ efflux events if the soils are disturbed and the carbon desorbed. These findings can be used to improve soil CO_2_ efflux estimates needed for forest carbon accounting.

## Electronic supplementary material

Below is the link to the electronic supplementary material.

Supplementary material 1 (DOCX 32 kb)
