# Supplementary material for: Forest soil CO2 efflux models improved by incorporating topographic controls on carbon content and sorption capacity of soils
Source: Biogeochemistry. 2016 Aug 19;129(3):307–23. doi: 10.1007/s10533-016-0233-5 (PMC7175660; doi:10.1007/s10533-016-0233-5)
Supplement: Supplementary file 1 — Supplementary material 1 (DOCX 32 kb) [file 10533_2016_233_MOESM1_ESM.docx]

Table S1. CO_2_ efflux measurements (μmol m^-2^ s^-1^).

| Date | T15 CR | T15 SH | T15 FS | T15 TS | T15 OW | T15 IW | T15 IW | T35 SH | T35 FS | T35 TS | T35 OW | T35 IW |
| --- | --- | --- | --- | --- | --- | --- | --- | --- | --- | --- | --- | --- |
| 4/8/2005 | 0.675 |  |  |  |  |  |  |  |  |  |  |  |
| 4/9/2005 | 0.728 | 0.956 |  |  |  |  |  | 0.536 | 1.360 |  |  |  |
| 4/10/2005 | 0.418 | 2.100 |  |  |  |  |  | 0.262 | 1.053 | 1.016 |  |  |
| 4/11/2005 | 0.266 | 1.424 |  |  |  |  |  | 0.173 | 0.725 | 0.524 |  |  |
| 4/12/2005 | 0.875 | 1.143 |  |  |  |  |  | 0.786 | 0.862 | 0.683 |  |  |
| 4/13/2005 | 0.639 | 0.917 |  |  | 0.497 |  |  | 0.740 | 0.699 | 0.451 |  |  |
| 4/14/2005 | 0.609 | 1.250 | 1.227 |  | 0.214 |  |  | 0.800 | 0.605 | 0.354 |  | 0.787 |
| 4/15/2005 | 0.963 | 1.368 | 1.470 |  | 0.643 |  |  | 0.957 | 1.066 | 1.361 |  | 0.997 |
| 4/16/2005 | 0.614 | 0.937 | 1.118 |  | 0.497 | 0.130 | 0.258 | 1.193 | 1.144 | 1.326 |  | 3.469 |
| 4/17/2005 | 1.344 | 1.776 | 3.545 |  | 0.522 | 0.199 | 0.365 | 1.192 | 1.111 | 1.145 |  | 0.994 |
| 4/18/2005 | 0.725 | 1.463 | 2.048 |  | 0.719 | 0.040 | 0.322 | 1.474 | 1.537 | 2.484 |  | 3.150 |
| 4/19/2005 | 1.402 | 2.159 | 3.341 | 2.675 | 1.458 | 0.435 | 0.766 | 1.255 | 1.199 | 1.895 | 2.057 | 2.325 |
| 4/20/2005 | 0.741 | 1.113 | 1.411 | 1.160 | 1.075 | 0.126 | 0.464 | 1.801 | 1.345 | 1.027 | 2.292 | 2.823 |
| 4/21/2005 | 1.147 | 1.39 | 1.621 | 1.224 | 0.997 | 0.210 | 0.501 | 0.840 | 0.853 | 0.980 | 1.566 | 1.473 |
| 4/22/2005 | 0.755 | 0.805 | 1.066 | 1.128 | 0.597 | 0.170 | 0.468 | 1.180 | 1.053 | 0.552 | 1.909 | 1.507 |
| 4/23/2005 | 0.328 | 0.386 | 0.748 | 0.680 | 0.550 | 0.131 | 0.258 | 0.281 | 0.195 | 0.106 | 0.939 | 0.555 |
| 4/24/2005 | 0.392 | 0.419 | 0.611 | 0.589 | 0.454 | 0.101 | 0.081 | 0.646 | 0.633 | 0.275 | 1.234 | 0.872 |
| 4/25/2005 | 0.639 | 0.45 | 0.850 | 0.714 | 0.552 | 0.162 | 0.291 | 1.022 | 0.680 | 1.025 | 0.893 | 1.305 |
| 4/28/2005 | 0.770 | 0.746 | 0.742 | 0.800 | 0.739 | 0.087 | 0.209 | 0.862 | 0.715 | 1.432 | 3.215 | 1.874 |
| 5/1/2005 | 0.705 | 0.645 | 0.790 | 0.752 | 0.557 | 0.172 | 0.274 | 0.584 | 0.438 | 1.122 | 2.218 | 1.640 |
| 5/10/2005 | 1.613 | 2.299 | 2.228 | 2.658 | 2.895 | 0.350 | 0.584 | 2.874 | 2.059 | 4.548 | 7.713 | 5.736 |
| 5/11/2005 | 0.941 | 1.292 | 1.426 | 1.665 | 1.534 | 0.520 | 0.143 | 1.543 | 1.939 | 0.875 | 2.238 | 3.503 |
| 5/12/2005 | 0.663 | 0.566 | 0.666 | 0.486 | 0.907 | 0.145 | 0.064 | 1.652 | 1.332 | 1.480 | 2.642 | 1.932 |
| 5/19/2005 | 1.248 | 2.329 | 2.166 | 2.156 | 1.887 | 0.652 | 0.296 |  |  |  | 2.779 | 2.642 |
| 5/20/2005 | 1.090 | 1.413 | 1.424 | 1.608 | 1.349 | 0.335 | 0.482 |  |  | 4.801 | 4.980 | 3.973 |
| 5/21/2005 | 1.545 | 2.183 | 2.100 | 2.145 | 2.023 | 0.969 | 1.291 |  |  | 2.544 | 3.739 | 3.782 |
| 5/28/2005 | 2.06 | 2.75 | 2.448 | 2.690 | 2.621 | 0.614 | 0.496 |  |  | 5.018 | 6.630 | 5.177 |
| 5/29/2005 | 1.692 | 2.61 | 2.020 | 2.769 | 2.063 | 0.722 | 0.687 |  |  |  | 5.966 | 3.929 |
| 5/30/2005 | 1.887 | 2.905 | 2.284 | 2.975 | 2.374 | 0.692 | 0.539 |  |  | 4.105 | 5.523 | 5.062 |
| 6/7/2005 | 3.236 | 4.003 | 3.357 | 4.032 | 3.295 | 1.299 | 2.365 |  |  | 6.645 | 8.398 | 6.359 |
| 6/8/2005 | 2.692 | 3.413 | 2.859 | 3.937 | 3.327 | 0.994 | 0.973 |  |  | 5.321 | 5.962 | 6.497 |
| 6/9/2005 | 4.130 | 5.499 | 4.903 | 6.564 | 4.928 | 1.249 | 1.955 | 5.262 | 3.452 | 8.455 | 12.519 | 8.815 |
| 7/5/2005 | 3.128 | 3.487 | 3.140 | 4.243 | 3.668 | 1.504 | 3.103 | 7.068 | 3.498 | 5.153 | 7.479 | 7.269 |
| 7/6/2005 | 2.923 | 3.588 | 3.424 | 4.623 | 3.690 | 1.498 | 5.051 | 5.464 | 2.673 | 5.155 | 9.929 | 6.817 |
| 7/7/2005 | 3.070 | 3.517 | 2.984 | 4.082 | 3.876 | 1.660 |  | 6.015 | 2.898 | 5.496 | 13.36 | 7.046 |
| 7/23/2005 | 1.546 | 1.279 | 2.464 | 2.564 | 3.525 | 1.901 |  | 3.122 | 1.454 | 3.229 | 8.451 |  |
| 7/24/2005 | 1.798 | 1.833 | 2.779 | 3.516 | 5.429 | 2.285 |  | 3.537 | 1.575 | 3.891 | 12.816 | 9.268 |
| 7/25/2005 | 1.506 | 1.367 | 2.567 | 2.802 | 3.864 | 1.881 |  | 3.008 | 1.450 | 5.683 | 12.215 | 9.650 |
| 8/9/2005 |  |  |  |  |  |  |  | 6.230 | 3.406 | 7.060 |  |  |
| 8/10/2005 |  |  |  |  |  |  |  | 6.884 | 3.693 | 8.055 |  |  |
| 8/11/2005 |  |  |  |  |  |  |  | 4.857 | 2.800 | 13.163 |  |  |
| 8/23/2005 |  |  |  |  |  |  |  | 2.36 | 1.412 | 2.753 |  |  |
| 8/24/2005 |  |  |  |  |  |  |  | 2.643 | 1.522 | 3.464 |  |  |
| 8/25/2005 | 1.297 | 1.515 | 2.097 | 2.998 | 3.539 | 1.396 | 6.028 | 2.131 | 1.191 | 3.263 | 5.699 | 6.068 |
| 9/14/2005 | 2.434 | 2.407 | 2.811 | 3.011 | 4.994 | 1.740 | 5.245 | 4.339 | 2.384 | 4.219 |  |  |
| 9/15/2005 | 1.890 | 1.660 | 2.044 | 1.952 | 2.721 | 1.249 | 4.212 | 4.451 | 1.833 | 3.510 |  |  |
| 9/16/2005 | 2.077 | 1.382 | 2.145 | 2.383 | 2.867 | 1.733 | 7.826 | 3.965 | 1.427 | 3.287 |  |  |
| 9/20/2005 | 2.879 | 3.100 | 2.889 | 2.934 | 3.251 | 1.813 | 3.896 | 4.268 | 2.478 | 4.540 |  |  |
| 9/23/2005 |  |  |  |  |  |  | 4.158 | 3.813 | 2.445 | 3.649 | 7.288 | 7.201 |
| 9/26/2005 |  |  |  |  |  |  | 4.086 | 5.285 | 2.637 | 4.155 | 7.792 | 7.571 |
| 9/29/2005 |  |  |  |  |  |  | 1.099 | 3.331 | 1.840 | 4.463 | 3.108 | 5.123 |
| 10/5/2005 | 4.111 | 4.952 | 4.793 | 5.344 | 5.277 | 2.481 | 3.938 | 6.632 | 4.424 | 7.981 |  |  |
| 10/8/2005 | 1.063 | 0.961 | 1.689 | 1.066 | 1.203 | 1.071 | 1.693 | 1.065 | 0.798 | 0.689 |  |  |
| 10/12/2005 | 1.964 | 2.182 | 2.610 | 3.058 | 3.056 | 1.293 | 2.304 | 2.224 | 1.825 | 3.634 |  |  |
| 10/16/2005 | 1.678 | 1.650 | 2.209 | 2.972 | 2.626 | 1.025 | 1.032 | 2.036 | 1.903 | 2.120 | 6.536 | 3.069 |
| 10/18/2005 | 1.575 | 0.665 | 1.018 | 1.084 | 1.941 | 1.547 | 1.906 | 2.495 | 1.655 | 3.560 | 8.208 | 3.711 |
| 10/21/2005 | 1.220 | 0.922 | 1.658 | 2.118 | 3.797 | 0.849 | 1.733 | 1.554 | 0.970 | 2.735 | 3.312 | 3.491 |
| 10/25/2005 | 0.978 | 1.043 | 1.151 | 1.518 | 1.814 | 0.618 | 0.983 | 1.377 | 1.084 | 1.062 | 2.345 | 1.613 |
| 11/28/2005 | 0.178 | 0.636 | 0.767 | 0.401 | 1.984 | 0.134 | 0.106 | 0.391 | 0.374 | 1.050 | 0.728 | 0.933 |
| 5/16/2010 |  | 2.351 | 1.682 | 1.870 | 1.732 | 1.242 | 1.398 | 3.521 | 2.609 | 2.833 | 5.323 | 4.483 |
| 5/18/2010 |  | 2.349 | 1.604 | 2.369 | 2.025 | 1.195 | 1.632 | 4.383 | 3.197 | 5.547 | 5.882 | 6.836 |
| 6/16/2010 |  | 0.412 | 3.856 | 4.293 |  | 0.466 | 1.317 | 6.525 | 7.243 | 3.607 | 6.932 | 7.783 |
| 6/17/2010 |  | 4.235 | 4.858 | 4.704 | 4.510 | 1.223 | 2.568 | 9.085 | 7.383 | 10.130 | 12.744 | 10.095 |
| 6/19/2010 |  | 4.996 | 4.842 | 5.137 | 6.011 | 2.538 |  |  |  |  |  |  |
| 6/20/2010 |  | 4.515 | 3.377 | 4.641 | 4.124 | 2.681 |  | 7.367 | 6.281 | 8.566 | 11.540 | 17.637 |
| 6/21/2010 |  |  |  |  |  |  |  | 7.462 | 3.252 | 9.136 | 7.837 | 10.193 |
| 6/23/2010 |  |  |  |  |  |  |  | 9.713 | 6.054 | 10.270 | 14.29 | 10.728 |
| 7/13/2010 |  |  |  |  |  |  |  | 9.178 | 6.155 | 12.716 | 18.421 | 11.511 |
| 7/14/2010 |  | 4.494 | 4.498 | 4.357 | 5.696 | 2.407 | 0.687 |  |  |  |  |  |
| 7/15/2010 |  | 4.817 | 4.809 | 5.465 | 6.610 | 2.259 | 1.864 |  |  |  |  |  |
| 7/16/2010 |  |  |  |  |  |  |  | 9.179 | 6.195 | 9.085 | 13.291 | 11.547 |
| 7/17/2010 |  | 3.999 | 3.920 | 4.827 | 2.934 | 2.586 | 3.096 | 8.519 | 5.688 | 15.560 | 13.528 | 10.609 |
| 7/19/2010 |  | 5.306 | 5.113 | 6.137 | 1.420 | 2.657 | 5.042 |  |  |  |  |  |
| 7/20/2010 |  |  |  |  |  |  |  | 9.974 | 6.282 | 25.355 | 17.966 | 9.860 |
| 7/21/2010 |  |  |  |  |  |  |  | 11.676 | 8.242 | 24.325 | 17.034 | 12.234 |
| 7/22/2010 |  |  | 4.735 | 4.388 | 6.663 | 3.460 | 4.866 |  |  |  |  |  |
| 7/24/2010 |  | 5.106 | 4.220 | 4.624 | 5.666 | 2.552 | 8.029 | 8.935 | 6.449 | 23.730 | 17.119 | 11.449 |
| 7/25/2010 |  |  |  |  |  |  |  | 9.719 | 6.226 | 22.785 | 13.915 | 18.962 |
| 7/26/2010 |  | 4.820 | 6.079 | 3.947 | 6.039 | 3.098 |  | 7.709 | 5.117 | 14.010 | 9.886 | 11.911 |
| 7/28/2010 |  | 4.570 | 5.731 | 6.335 | 6.314 | 3.552 | 6.567 |  |  |  |  |  |
| 8/18/2010 |  | 3.230 | 3.601 | 2.685 | 3.184 | 1.716 | 5.435 | 6.300 | 5.298 | 11.690 | 12.334 | 7.584 |
| 8/19/2010 |  |  |  |  |  |  |  | 9.076 | 7.276 | 21.485 | 9.158 | 11.035 |
| 8/20/2010 |  | 3.456 | 4.645 | 3.086 | 5.502 | 2.474 | 10.310 | 6.484 | 3.729 | 14.769 | 11.936 | 11.368 |
| 8/21/2010 |  | 5.725 | 5.750 | 5.973 | 6.539 | 1.052 | 2.962 | 7.772 | 7.334 | 13.379 | 15.538 | 10.117 |
| 8/22/2010 |  | 5.509 | 5.918 | 4.293 | 5.104 | 1.423 | 2.486 | 7.800 | 8.729 | 12.510 | 8.474 | 2.950 |
| 8/23/2010 |  | 5.121 | 5.200 | 4.566 | 5.256 | 3.358 | 2.612 | 8.640 | 5.659 | 14.762 | 13.041 | 10.872 |
| 8/24/2010 |  | 3.862 | 9.814 | 4.447 | 8.884 | 2.676 | 3.458 | 7.103 | 5.016 | 19.218 | 10.500 | 18.099 |
